# HnRNP K mislocalisation in neurons of the dentate nucleus is a novel neuropathological feature of neurodegenerative disease and ageing

**DOI:** 10.1111/nan.12793

**Published:** 2022-02-09

**Authors:** Rahul Sidhu, Ariana Gatt, Pietro Fratta, Tammaryn Lashley, Alexander Bampton

**Affiliations:** ^1^ The Queen Square Brain Bank for Neurological Disorders, Department of Clinical and Movement Neuroscience UCL Queen Square Institute of Neurology London UK; ^2^ Department of Neurodegenerative diseases UCL Queen Square Institute of Neurology London UK; ^3^ Department of Neuromuscular diseases UCL Queen Square Institute of Neurology London UK

**Keywords:** Alzheimer's disease, dentate nucleus, frontotemporal dementia, hnRNP K

## Abstract

Nuclear depletion and cytoplasmic mislocalisation of the RNA‐binding protein heterogeneous ribonucleoprotein K (hnRNP K) within pyramidal neurons of the frontal cortex have been shown to be a common neuropathological feature in frontotemporal lobar degeneration (FTLD) and elderly control brain. Here, we describe a second neuronal subtype vulnerable to mislocalisation within the dentate nucleus of the cerebellum. In contrast to neurons within the cerebellar cortex that typically exhibited normal, nuclear staining, many neurons of the dentate nucleus exhibited striking mislocalisation of hnRNP K to the cytoplasm within neurodegenerative disease brain. Mislocalisation frequency in this region was found to be significantly higher in both FTLD‐TDP A and Alzheimer's disease (AD) brain than in age‐matched controls. However, within control (but not disease) subjects, mislocalisation frequency was significantly associated with age‐at‐death with more elderly controls typically exhibiting greater levels of the pathology. This study provides further evidence for hnRNP K mislocalisation being a more anatomically diverse pathology than previously thought and suggests that potential dysfunction of the protein may be more broadly relevant to the fields of neurodegeneration and ageing.

## Graphical abstract

HnRNP K mislocalisation from the nucleus to the cytoplasm is a commonly observed neuropathological feature in neurons of the dentate nucleus in frontotemporal lobar degeneration (FTLD) and Alzheimer's disease (AD) brain. In keeping with previous findings within pyramidal neurons of the cortex, hnRNP K mislocalisation correlates with age in neurologically normal controls. Hence, hnRNP K mislocalisation and dysfunction is a more neuroanatomically widespread phenomenon than first appreciated and is potentially mechanistically relevant to the wider field of dementia.

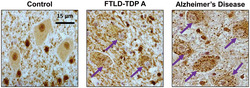

**Key Points**

- Neurons within the dentate nucleus of the cerebellum are another neuronal subtype vulnerable to hnRNP K mislocalisation.
- hnRNP K mislocalisation in neurons of the dentate nucleus occurs more frequently in neurodegenerative disease brain than in age‐matched controls.
- hnRNP K mislocalisation correlates with age at death in control cases.

Heterogeneous ribonucleoprotein K (hnRNP K) is a member of the functionally versatile hnRNP family of RNA‐binding proteins. We have recently described a novel protein pathology whereby hnRNP K is depleted from the nucleus and mislocalised to the cytoplasm in pyramidal neurons of the frontal cortex [1]. This event was found to be more frequent in cases of neurodegenerative disease (frontotemporal lobar degeneration [FTLD]) than in age‐matched control subjects. Mislocalisation was also found to be positively associated with age at death in controls suggesting nuclear loss of hnRNP K in FTLD brain may constitute an advanced ageing phenotype. Cytoplasmic accumulation of hnRNP K is also a frequent neuropathological finding across several malignancies and has been observed in motor neurons subjected to osmotic stress [2, 3, 4].

HnRNP K mislocalisation in layers III and V pyramidal neurons is intriguing because these neurons are not typically associated with classical FTLD pathologies, and indeed, we previously demonstrated the mutual exclusivity of hnRNP K and TDP‐43/Tau pathologies in FTLD brain [1]. This prompted us to explore other brain regions and neuronal subtypes that may also exhibit hnRNP K mislocalisation but which are not usually implicated in neurodegeneration. Moreover, In addition to FTLD‐TDP subtypes and FTLD‐Tau, we also previously identified mislocalisation in aged controls which had certain degrees of underlying Alzheimer's disease (AD) pathology. We have included an AD cohort in this study to assess whether clinically confirmed cases with underlying AD pathology also showed hnRNP K mislocalisation, which could potentially highlight the importance of RNA‐binding proteins in the broader field of neurodegeneration regardless of the protein aggregates deposited.

The focus of this study was on the cerebellum where we investigated hnRNP K localisation within the dentate nucleus and the cerebellar cortex. We identify the dentate nucleus to be another region vulnerable to neuronal hnRNP K mislocalisation and report, by contrast, normal staining across the cerebellar cortex. We analysed two disease cohorts (FTLD‐TDP A and AD) and one age‐matched control cohort to assess the specificity of this pathology and an additional group of controls to investigate the role of normal ageing.

Immunohistochemical analysis was performed on age‐matched FTLD‐TDP A (*n* = 19, 65.9 ± 6.9 years), AD (*n* = 17, 63.2 ± 13.5 years) and control (*n* = 21. 62.7 ± 15.1 years; *n* = 32 total) cases according to standard procedures (Table S1). Once stained, eight random sample images (×20) of the dentate nucleus were acquired per case. Two assessors counted neurons within each image field exhibiting either normal (nuclear) or abnormal hnRNP K staining (nuclear loss and punctate cytoplasmic accumulation) blinded to disease status. Mislocalisation frequency was reported as the proportion (%) of all neurons exhibiting hnRNP K mislocalisation.

We found neurons within the dentate nucleus to exhibit distinctly different hnRNP K localisation profiles across the cohorts. Typical control subjects exhibited normal hnRNP K localisation with strong nuclear staining intensity and weaker cytoplasmic staining (Figure 1A), while FTLD/AD subjects typically exhibited an abnormal pattern of staining within these neurons consisting of hnRNP K nuclear depletion and granular cytoplasmic accumulation of the protein (Figure 1B,C).

**FIGURE 1:**
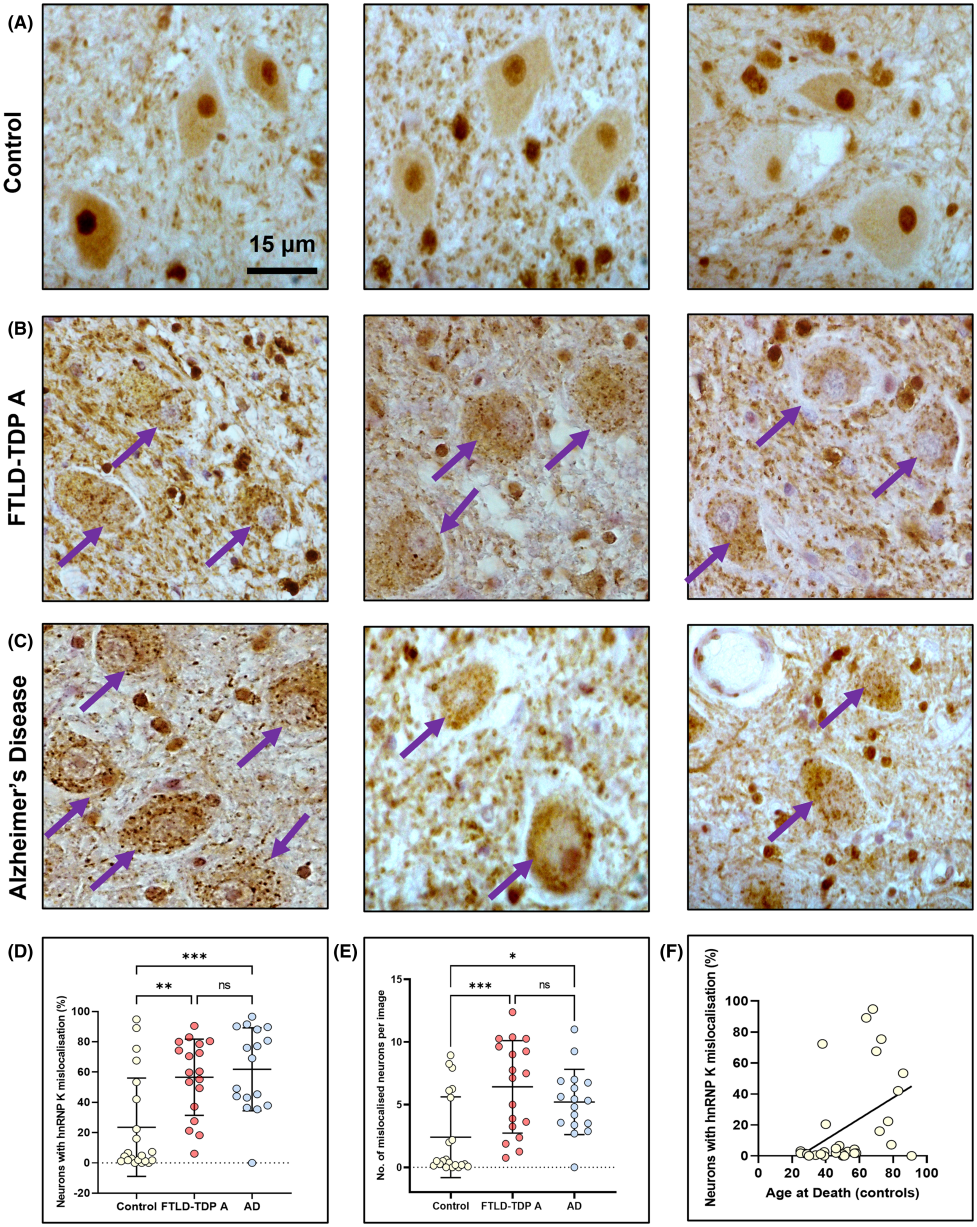
HnRNP K mislocalisation in neurons of the dentate nucleus. (A) Representative (×60) images of normal, nuclear localisation of hnRNP K in neurons of the dentate nucleus within three control subjects. (B) Representative images of abnormal, mislocalised neuronal staining of hnRNP K within three FTLD‐TDP A cases exhibiting distinct nuclear depletion and cytoplasmic puncta accumulation. (C) Representative images of hnRNP K mislocalisation in three Alzheimer's disease (AD) cases. Purple arrows indicate neurons with clear hnRNP K nuclear depletion. Scale bars are as indicated in first image. (D) Quantitation of hnRNP K mislocalisation expressed as proportion (%) of neurons with hnRNP K mislocalisation in age‐matched control (*n* = 21), FTLD‐TDP A (*n* = 18) and AD (*n* = 17) cohorts. (E) Equivalent quantitation and analysis expressing hnRNP K mislocalisation as number of neurons per image exhibiting mislocalisation. Individual data points indicate mean data from distinct cases. Error bars show mean ± SD. Ordinary one‐way ANOVA with Tukey's post hoc test; ^*^ *p* < 0.05, ^**^ *p* < 0.01, ^***^ *p* < 0.001. (F) HnRNP K mislocalisation in control subjects positively correlates with age at death (Pearson's *r* = 0.433, *p* = 0.011)

The frequency (%) of neuronal mislocalisation of hnRNP K was significantly higher within FTLD‐TDP A (*p* = 0.0026) and AD (*p* = 0.0004) groups versus age‐matched controls (Figure 1D). The same result was observed when comparing the number of mislocalised neurons per image with both FTLD‐TDP A (*p* = 0.0010) and AD (*p* = 0.0251) exhibiting more frequent mislocalised neurons than control subjects (Figure 1E). There was no difference in mislocalisation frequency, as defined by either metric, between familial and sporadic AD groups or between *C9orf72* mutation‐carrying and non‐C9orf72‐associated FTLD‐TDP A subjects (data not shown). By including an additional set of control subjects of varying ages (*n* = 31 total), we were able to investigate the relationship between hnRNP K mislocalisation and age. hnRNP K mislocalisation frequency (%) within neurons of the dentate neurons correlated with age at death (*r* = 0.433, *p* = 0.011) in the control cohort, but no such association was found within FTLD‐TDP A or AD groups (Figures 1D and S1A,B). Mislocalisation frequency was not associated with any other available demographic or clinical covariate including sex, brain weight, disease duration or post‐mortem delay (data not shown).

To determine whether hnRNP K mislocalisation in the dentate nucleus was related to mislocalisation in the cortex, we correlated our FTLD‐TDP A case‐matched scores from this cohort and our previous dataset [1]. We found a significant association (*r* = 0.520, *p* = 0.027) between case scores (*n* = 18) suggesting cases vulnerable to hnRNP K mislocalisation in one brain region are more susceptible to mislocalisation in another (Figure S1C).

By visual inspection, we observed neurons within the cerebellar cortex to exhibit a normal hnRNP K localisation profile irrespective of disease status or age in contrast with the dentate nucleus. Neurons within the molecular and granular layers as well as Purkinje cell bodies all exhibited strong and predominantly nuclear staining patterns (Figure S2).

These results bring us to three main conclusions. First, hnRNP K mislocalisation is not restricted to pyramidal neurons of the cortex or even indeed to anatomical regions most susceptible to neurodegeneration. It will be of mechanistic interest to delineate potential structural and/or functional similarities between vulnerable cell types. We note for example that both pyramidal neurons and neurons within the dentate nucleus are among the largest neurons within their respective brain regions and may well have similarly high energetic demands to sustain their metabolism. Characterisation studies aimed at identifying key links between neuronal subtypes that may include other morphological factors, neurotransmitter profiles or other shared histological features may shed light on why these neurons are especially susceptible to hnRNP K mislocalisation.

Second, hnRNP K pathology is more frequent in neurodegenerative disease than in age‐matched controls, and this is not limited to FTLD subjects that broaden the relevance of emerging hnRNP K pathobiology to the wider field of dementia. Third, as observed previously, hnRNP K mislocalisation in neurologically normal control subjects is more commonly observed in older individuals (>60 years), but no such relationship with age is observed in disease cohorts. It will be of special interest to determine which factors determine the predisposition to hnRNP K mislocalisation in different individuals and disease statuses at varying ages.

In summary, we believe these novel pathological insights to provide evidence for hnRNP K mislocalisation and dysfunction in neurodegeneration and ageing being a more widespread phenomenon across the brain than previously thought. One emerging mechanism of hnRNP dysfunction in neurodegenerative disease is the derepression of cryptic exons [5]. We have previously shown hnRNP K nuclear loss in neurons to be associated with inclusion of cryptic exons which may destabilise the transcript [1]. Future in situ hybridisation (ISH) investigations will shed light on whether neurons showing hnRNP K nuclear depletion in different brain regions harbour cryptic exons, offering a potentially functional read‐out of hnRNP K loss of function and neuronal toxicity.

## CONFLICT OF INTEREST

The authors declare no conflicts of interest.

## AUTHOR CONTRIBUTIONS

AB and TL conceptualised the study and accompanying experiments. RS performed the majority of the immunohistochemistry with assistance from AB. RS and AB were equally responsible for image acquisition, data collection and analysis. AB drafted the initial version of this manuscript with assistance from AG and TL. All authors approved the final version of this manuscript.

## ETHICS STATEMENT

Brains were donated from both the Queen Square Brain Bank (QSBB) and the Medical Research Council (MRC) Edinburgh Brain & Tissue Bank. Appropriate, informed consent was obtained from all donors. Ethical approval for this study was obtained from the NHS research ethics committee, and all laboratory work was performed under the human tissue authority (HTA) licence 12198.

### PEER REVIEW

The peer review history for this article is available at https://publons.com/publon/10.1111/nan.12793.

## Supporting information


**Figure S1.** HnRNP K mislocalisation does not significantly correlate with age at death in **a** FTLD‐TDP A or **b** Alzheimer's disease cases in contrast to control cases (fig 1f). **c** HnRNP K mislocalisation in neurons of the dentate nucleus significantly correlates with equivalent mislocalisation in the frontal cortex (Bampton et al, 2021) within case‐matched brains.

**Figure S2.** Normal hnRNP K localisation in neurons of the cerebellar cortex. Representative images of normal, predominantly nuclear hnRNP K staining in neurons of the cerebellum cortex in both **a** control and **b** neurodegenerative disease (FTLD/TDP A) brain.

**Table S1.** Cohort and clinical demographics. *Non‐age matched control subjects added to cohort to investigate the relationship between hnRNP K mislocalisation and ageing; AD ‐ Alzheimer's disease; CBD ‐ Corticobasal degeneration; FAD ‐ Familial Alzheimer's disease; FTD ‐ Frontotemporal dementia; FTLD ‐ Frontotemporal lobar degeneration; bvFTD ‐ Behavioural variant FTD; PCA ‐ Posterior cortical atrophy; PNFA ‐ Progressive non‐fluent primary aphasia variant FTD; MND ‐ Motor neuron disease.

## Data Availability

The data that support the findings of this study are available from the corresponding author upon reasonable request.
