# Supplementary figures and images for: HnRNP K mislocalisation in neurons of the dentate nucleus is a novel neuropathological feature of neurodegenerative disease and ageing
Source: Neuropathol Appl Neurobiol. 2022 Feb 9;48(4):e12793. doi: 10.1111/nan.12793 (PMC9208575; doi:10.1111/nan.12793)

**a**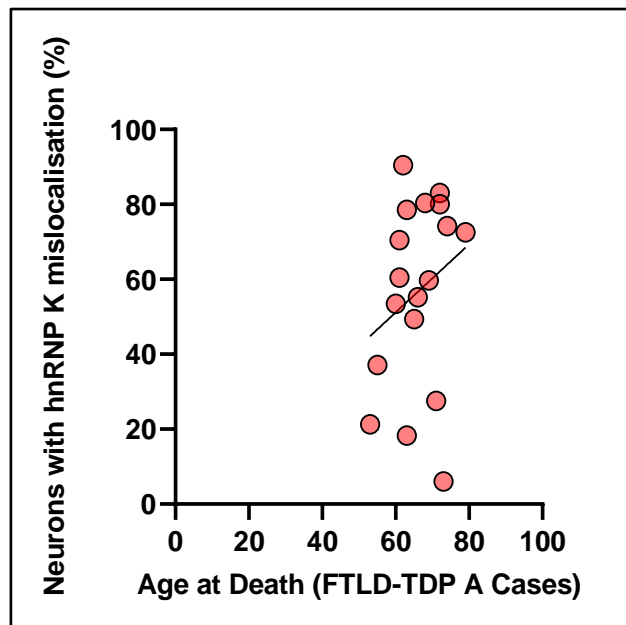**b**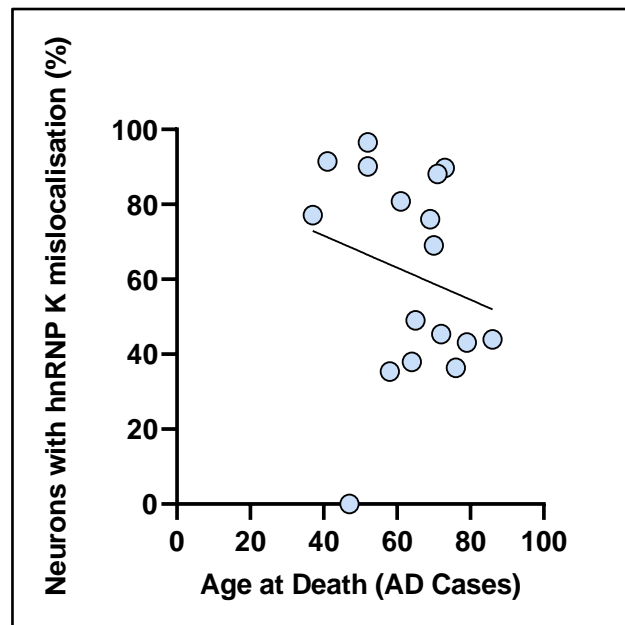**c**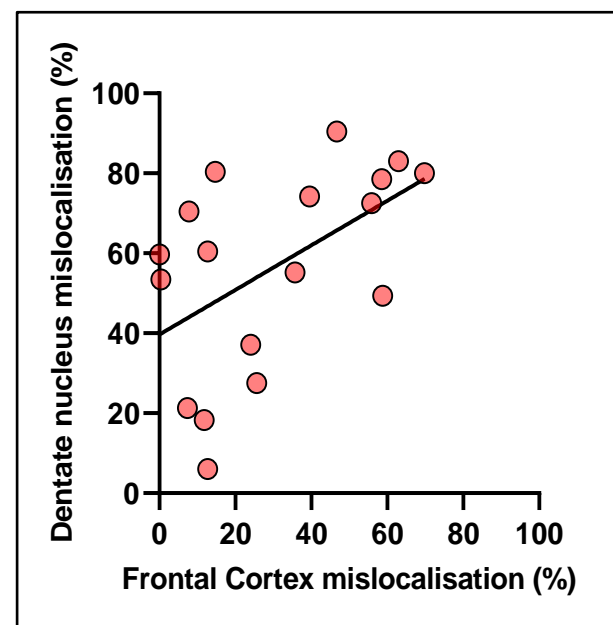

Supplement: Supplementary file 1 — Figure S1. HnRNP K mislocalisation does not significantly correlate with age at death in a FTLD‐TDP A or b Alzheimer's disease cases in contrast to control cases (fig 1f). c HnRNP K mislocalisation in neurons of the dentate nucleus significantly correlates with equivalent mislocalisation in the frontal cortex (Bampton et al, 2021) within case‐matched brains. [file NAN-48-e12793-s002.pdf]

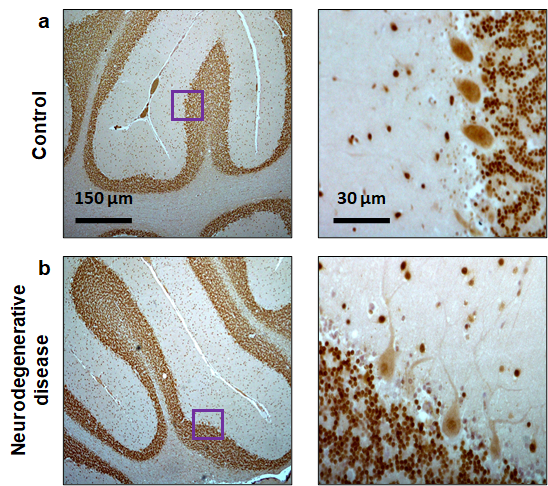

Supplement: Supplementary file 2 — Figure S2. Normal hnRNP K localisation in neurons of the cerebellar cortex. Representative images of normal, predominantly nuclear hnRNP K staining in neurons of the cerebellum cortex in both a control and b neurodegenerative disease (FTLD/TDP A) brain. [file NAN-48-e12793-s003.png]
